# A Role of Cholesterol in Modulating the Binding of α-Synuclein to Synaptic-Like Vesicles

**DOI:** 10.3389/fnins.2020.00018

**Published:** 2020-01-29

**Authors:** Wing K. Man, Alfonso De Simone, Joseph D. Barritt, Michele Vendruscolo, Christopher M. Dobson, Giuliana Fusco

**Affiliations:** ^1^Department of Chemistry, Centre for Misfolding Diseases, University of Cambridge, Cambridge, United Kingdom; ^2^Department of Life Sciences, Imperial College London, London, United Kingdom

**Keywords:** α-synuclein, synaptic vesicles, membrane interaction, cholesterol, nuclear magnetic resonance

## Abstract

α-Synuclein (αS) is a presynaptic protein whose aggregation is associated with Parkinson’s disease (PD). Although the physiological function of αS is still unclear, several lines of evidence indicate that this protein may play a role in the trafficking of synaptic vesicles (SVs) during neurotransmitter release, a task associated with its ability to bind SVs and promote their clustering. It is therefore crucial to identify the cellular factors that modulate this process. To address this question, using nuclear magnetic resonance (NMR) spectroscopy we have characterized the role of cholesterol, a major component of the membrane of SVs, in the binding of αS with synaptic-like vesicles. Our results indicate that cholesterol can act as a modulator of the overall affinity of αS for SVs by reducing the local affinity of the region spanning residues 65–97 in the non-amyloid-β component (NAC) of the protein. The increased population of bound states that expose the region 65–97 to the solvent was found to induce stronger vesicle-vesicle interactions by αS. These results provide evidence that cholesterol modulates the clustering of synaptic vesicles induced by (α)S, and supports the role of the disorder-to-order equilibrium of the NAC region in the modulation of the biological properties of the membrane-bound state of αS.

## Introduction

α-Synuclein (αS) is a 140-residue protein primarily expressed in neuronal cells, where it localizes predominantly at the pre-synaptic termini (Jakes et al., 1994). The aggregation of αS is linked with Parkinson’s disease (PD), with its aggregates being a major component of Lewy bodies in PD patients (Uversky and Eliezer, 2009; Luk et al., 2012; Lashuel et al., 2013; Chiti and Dobson, 2017; Fusco et al., 2017). Genetic links also exist between αS and PD, with point mutations, duplication and triplications in the αS encoding gene being associated with familial forms of the disease (Polymeropoulos et al., 1997; Kruger et al., 1998; Singleton et al., 2003; Zarranz et al., 2004; Appel-Cresswell et al., 2013; Lesage et al., 2013). αS aggregates have been found in other neurodegenerative disorders, including Alzheimer’s disease (Lewis et al., 2010), dementia with Lewy bodies (Galvin et al., 1999), multiple system atrophy (Spillantini et al., 1998), and other synucleinopathies (Chiti and Dobson, 2017). Despite the general consensus on the pathological relevance of αS aggregation, the function of this protein remains highly debated (Burre, 2015). A number of experimental observations indicate a physiological interaction between αS and synaptic vesicles (SVs), which has been associated with the regulation of SVs trafficking at the synaptic termini, including the assistance of the formation of the SNARE complex during neurotransmitter releases (Burre et al., 2010, 2014). It has also been suggested that αS might be involved in the maintenance of pools of SVs at the synapses (Cooper et al., 2006; Wislet-Gendebien et al., 2006; Auluck et al., 2010; Nemani et al., 2010; Vargas et al., 2014), a role that is associated with its ability to mediate interactions between SVs (Gitler et al., 2008; Soper et al., 2008; Diao et al., 2013; Fusco et al., 2016b). A common characteristics of the majority of the putative functions so far proposed for αS – including SV trafficking (Wislet-Gendebien et al., 2006; Gitler et al., 2008; Soper et al., 2008; Auluck et al., 2010; Burre et al., 2010, 2014; Diao et al., 2013), ER-to-Golgi vesicle trafficking (Cooper et al., 2006; Gitler et al., 2008), mitochondrial binding (Zigoneanu et al., 2012; Maltsev et al., 2013; Menges et al., 2017) – involves the binding with biological membranes (Zigoneanu et al., 2012; Maltsev et al., 2013; Snead and Eliezer, 2014). Indeed, the partition between membrane bound and unbound states of αS appears to be highly regulated *in vivo* (Lee et al., 2002) and influences its aggregation propensity (Perrin et al., 2001; Necula et al., 2003; Sharon et al., 2003; Zhu and Fink, 2003; Auluck et al., 2010; Comellas et al., 2012).

**FIGURE 1:**
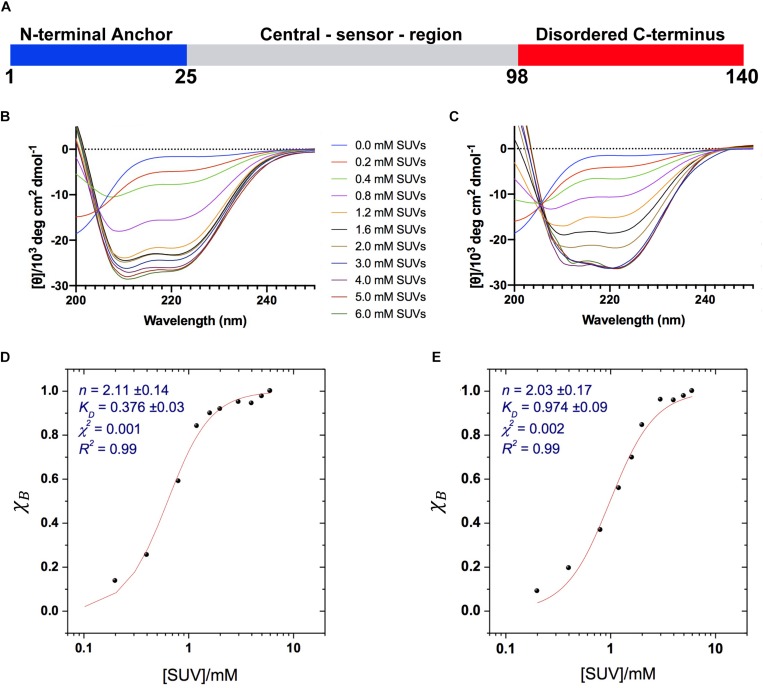
Binding of α*S* to acidic SUVs. **(A)** Three regions of αS were found to have specific structural and dynamical properties at the surface of SUV-0% (Fusco et al., 2014). These include the N-terminal anchor (residues 1–25, blue), whose ssNMR resonances spanning the region 6–25 were previously assigned (Fusco et al., 2014), the central sensor region (residues 26–97, gray), and the C-terminal domain (residues 98–140, red) remaining essentially unbound and disordered at the membrane surface. **(B–E)** CD measurements of αS binding to acidic SUV-0% **(B,D)** and SUV-31% **(C,E)**. In all measurements the concentration of αS was kept constant at 10 μM whereas the concentrations of SUV-0% and SUV-31% were calculated by considering exclusively the DOPE:DOPS:DOPC component in both types of vesicles. **(B,C)** CD titration in 20 mM buffer, pH 6.0 measured at 10°C for SUV-0% **(B)** and SUV-31% **(C)**. **(D,E)** Fitting of the CD titrations following the signal at 222 nm, [θ]_222 nm_ for SUV-0% **(D)** and SUV-31% **(E)**. The Hill equation was used to account for both binding constant *K*_*D*_ and the cooperativity, which is fitted with the Hill coefficient *n*.

Understanding the mechanism of interaction of αS with lipid membranes and the conformational properties of its bound state is therefore crucial to clarify the balance between functional and dysfunctional forms of this protein. The significant levels of structural disorder in both bound and unbound states, however, pose significant experimental challenges in characterizing this mechanism. Upon lipid membrane binding, αS undergoes a transition from an intrinsically disordered protein to a partially α-helical state that retains a significant level of structural disorder (Eliezer et al., 2001; Ulmer and Bax, 2005; Bodner et al., 2009; Maltsev et al., 2012). The α-helical segments in the membrane-bound αS are promoted by seven imperfect sequence repeats in the region 1–90 that encode for amphipathic α-helices (Eliezer et al., 2001). The modular organization of these repeats enables the binding of αS with a variety of lipid membranes, ranging from lipid micelles to lipid vesicles and cellular membranes (Ulmer and Bax, 2005; Ulmer et al., 2005; Jao et al., 2008; Bodner et al., 2009), and via a multiplicity of distinct binding modes (Bodner et al., 2009), including a broken (Ulmer and Bax, 2005; Ulmer et al., 2005) and an extended α-helix (Jao et al., 2008; Lokappa and Ulmer, 2011; Cheng et al., 2013). Several studies have indicated that the binding of αS involves an initial membrane interaction by the N-terminal 25 residues in an α-helical conformation (Fusco et al., 2016a) and the cooperative propagation of the α-helical structure throughout the central region (residues 26–97), while the C-terminal region of the protein remains essentially unbound to the membrane surface (Figure 1A; Bodner et al., 2009; Fusco et al., 2014). Membrane binding by αS can also be influenced by a variety of factors, including the properties of the membrane such as charge, defects, curvature, lipid rafts, and the properties of αS such as point mutations (Bodner et al., 2010; Fusco et al., 2016b) and post-translational modifications (Fauvet et al., 2012). The sensitivity of the binding modes to even relatively minor external factors has therefore prompted a number of studies to investigate the lipid membrane interaction by αS under conditions that reproduce as closely as possible the physiological context in which αS is present.

In this work, we characterized the role of cholesterol in the interaction between αS and synaptic-like vesicles using nuclear magnetic resonance (NMR) spectroscopy and other biophysical techniques. Cholesterol is a key component of SVs, accounting of 31% w/w of their total membrane composition (Takamori et al., 2006). Our study found that the overall membrane-binding affinity of αS for synaptic-like vesicles is substantially reduced in the presence of cholesterol, with major effects occurring primarily in the region spanning residues 65–97. Our data are discussed in view of the mechanism by which αS can induce vesicle-vesicle interactions (Fusco et al., 2016b), a property that has been associated with the maintenance of SVs homeostasis during neurotransmitter release.

## Materials and Methods

### αS Purification

α-Synuclein was expressed and purified as previously described (Fusco et al., 2014). Briefly the protein was expressed in *E. coli* using plasmid pT7-7. After transforming in BL21 (DE3)-gold cells (Agilent Technologies, Santa Clara, United States), uniformly ^15^N and/or ^13^C labeled αS variants were obtained by growing the bacteria in isotope-enriched M9 minimal media containing 1 g^.^L^–1^ of ^15^N ammonium chloride, 2 g^.^L^–1^ of ^13^C-glucose (Sigma-Aldrich, St. Louis, United States). The growth was obtained at 37°C under constant shaking at 250 rpm and supplemented with 100 μg^.^ml^–1^ ampicillin to an OD600 of 0.6. The expression was induced with 1 mM isopropyl β-D-1-thiogalactopyranoside (IPTG) at 37°C for 4 h, and the cells were harvested by centrifugation at 6200 *g* (Beckman Coulter, Brea, United States). The cell pellets were resuspended in lysis buffer (10 mM Tris–HCl pH 8, 1 mM EDTA, and EDTA-free complete protease inhibitor cocktail tablets obtained from Roche, Basel, Switzerland) and lysed by sonication. The cell lysate was centrifuged at 22,000 *g* for 30 min to remove cell debris. In order to precipitate the heat-sensitive proteins, the supernatant was then heated for 20 min at 70°C and centrifuged at 22,000 *g*. Subsequently streptomycin sulfate was added to the supernatant to a final concentration of 10 mg^.^ml^–1^ to stimulate DNA precipitation. The mixture was stirred for 15 min at 4°C followed by centrifugation at 22,000 *g*. Then, ammonium sulfate was added to the supernatant to a concentration of 360 mg^.^ml^–1^ in order to precipitate the protein. The solution was stirred for 30 min at 4°C and centrifuged again at 22,000 *g*. The resulting pellet was resuspended in 25 mM Tris–HCl, pH 7.7 and dialyzed against the same buffer in order to remove salts. The dialyzed solutions were then loaded onto an anion exchange column (26/10 Q sepharose high performance, GE Healthcare, Little Chalfont, United Kingdom) and eluted with a 0–1 M NaCl step gradient, and then further purified by loading onto a size exclusion column (Hiload 26/60 Superdex 75 preparation grade, GE Healthcare, Little Chalfont, United Kingdom). All the fractions containing the monomeric protein were pooled together and concentrated by using Vivaspin filter devices (Sartorius Stedim Biotech, Göttingen, Germany). The purity of the aliquots after each step were analyzed by SDS-PAGE and the protein concentration was determined from the absorbance at 275 nm using an extinction coefficient of 5600 M^–1^ cm^–1^.

### Preparation of SUVs

We prepared two types of small unilamellar vesicles (SUVs). One SUV type was made using a mixture of DOPE:DOPS:DOPC lipids (Avanti Polar Lipids Inc., Alabaster, United States) in a ratio of 5:3:2 w/w. A second type of vesicles was prepared using DOPE:DOPS:DOPC lipids (5:3:2 w/w) and non-esterified cholesterol, the latter amounting to 31% w/w of the total mixture to mimic its natural abundance in the membrane component of SVs (Takamori et al., 2006). We denoted the two vesicle types as SUV-0% and SUV-31%. These SUVs were prepared from chloroform solutions as described previously (Bodner et al., 2009; Fusco et al., 2014). Briefly, the lipid mixture was evaporated under a stream of nitrogen gas and then dried thoroughly under vacuum to yield a thin lipid film. The dried thin film was then re-hydrated by adding aqueous buffer (20 mM sodium phosphate, pH 6.0) at a concentration of 10 mg^.^ml^–1^ (1.5%) and subjected to vortex mixing. In all NMR experiments described in this paper SUVs were obtained by using several cycles of freeze-thawing and sonication until the mixture became clear (Bodner et al., 2009; Fusco et al., 2014) and followed by a step of extrusion through membranes using pore diameters of 50 nm (Avanti Polar Lipids, Inc.). All SUVs were controlled with dynamic light scattering (DLS) in order to obtain vesicles with the same average size.

### Chemical Exchange Saturation Transfer (CEST) Experiments

Chemical Exchange Saturation Transfer (CEST) measurements in solution NMR (Milojevic et al., 2007; Huang et al., 2008; Fawzi et al., 2011; Vallurupalli et al., 2012; Fusco et al., 2014; Romano et al., 2018) probed the equilibrium between membrane unbound and membrane bound states of αS *via* direct detection of saturation in the resonances of the unbound state. In studying αS-SUV interactions, CEST shows higher sensitivity than measurements based on the signal attenuation in HSQC spectra, and enables measurements at low lipid:protein ratios to minimize αS or lipid aggregation (Fusco et al., 2014). Moreover, CEST signals are directly sensitive to the interaction between αS and the membrane surface and disclose the interference from additional factors that can contribute to the transverse relaxation rates of the protein resonances (Milojevic et al., 2007; Huang et al., 2008; Fawzi et al., 2011; Vallurupalli et al., 2012). CEST was carried out at 10°C on a sample composed of αS (300 μM) incubated with SUV-31% at a concentration of 0.6 mg/ml in 20 mM sodium phosphate buffer at pH 6.0, and using a Bruker spectrometer operating at ^1^H frequencies of 700 MHz equipped with triple resonance HCN cryo-probe. These experiments were based on ^1^H-^15^N HSQC spectra by applying constant wave saturation in the ^15^N channel. Assignment of the solution NMR resonances was obtained from our previous studies (Fusco et al., 2014) and controlled with a series of 3D spectra by following a published protocol (Fusco et al., 2012). Since we aimed at probing the exchange between monomeric αS (having sharp resonances) and αS bound to SUVs (having significantly broader resonances), a series of large offsets was employed (−28, −21, −14, −9, −5, −3, −1.5, 0, 1.5, 3, 5, 9, 14, 21, and 28 kHz), resulting in CEST profiles of symmetrical shapes (Huang et al., 2008; Fawzi et al., 2011; Fusco et al., 2014). An additional spectrum, saturated at −100 kHz, was recorded as a reference. The CEST experiments were measured using a data matrix consisting of 2048 (*t*_2_, ^1^H) × 220 (*t*_1_, ^15^N) complex points.

### Transverse Relaxation Experiments

Standard pulse sequences were used for T_2_ experiments (Farrow et al., 1994), including the watergate sequence (Piotto et al., 1992) to improve water suppression. *T*_2_ values were obtained by fitting single exponential decays to the experimental data; the fitting of experimental data and the error analyses were performed with the program SPARKY. Relaxation was measured at 10°C on a sample composed of αS (300 μM) incubated with SUV-31% at a concentration of 0.6 mg/ml and using a Bruker spectrometer operating at ^1^H frequencies of 700 MHz equipped with triple resonance HCN cryo-probe. Assignment of the solution NMR resonances was obtained from our previous studies (Fusco et al., 2014) and controlled with a series of 3D spectra by following a published protocol (Fusco et al., 2012).

### Dynamic Light Scattering (DLS)

Dynamic Light Scattering measurements of vesicle size distributions were performed using a Zetasizer Nano ZSP instrument (Malvern Instruments, Malvern, United Kingdom) with backscatter detection at a scattering angle of 173°. The viscosity (0.8882 cP) and the refractive index (1.330) of water were used as parameters for the buffer solution, and the material properties of the analyte were set to those of the lipids (absorption coefficient of 0.001 and refractive index of 1.440). SUVs were used at a concentration of 0.05% in these measurements and the experiments were performed at 25°C. The acquisition time for the collection of each dataset was 10 sec and accumulation of the correlation curves was obtained using 10 repetitions. Each measurement was repeated 10 times to estimate standard deviations and average values of the centers of the size distributions. Samples used in DLS measurements were incubated, with or without αS molecules for 1 h at 298 K using freshly prepared samples of SUV-0% and SUV-31% at a concentration of 0.05%. The concentrations of SUV-0% and SUV-31% were calculated by considering exclusively the DOPE:DOPS:DOPC component in both types of vesicles. Moreover, in order to compensate for the 2.6 times difference in *K*_*D*_, the concentration of αS was 77 μM and 200 μM when incubated with SUV-0% and SUV-31%, respectively.

### Circular Dichroism Analysis of αS in the Presence of Different Concentrations of SUVs

Circular Dichroism (CD) measurements were made at 10°C. CD samples were prepared in 20 mM sodium phosphate buffer at pH 6.0, by using a constant concentration of αS (10 μM) and variable concentrations of SUV-0% and SUV-31%. The SUV concentrations were calculated by considering exclusively the DOPE:DOPS:DOPC component in both types of vesicles. Far-UV CD spectra were recorded on a JASCO J-810 equipped with a Peltier thermally controlled cuvette holder. Quartz cuvettes with path lengths of 1 mm were used, and CD spectra were obtained by averaging ten individual spectra recorded between 250 nm and 200 nm with a bandwidth of 1 nm, a data pitch of 0.2 nm, a scanning speed of 50 nm/min and a response time of 4 s. Each value of the CD signal intensity reported at 222 nm corresponds to the average of ten measurements. For each protein sample, the CD signal of the buffer used to solubilize the protein was recorded and subtracted from the CD signal of the protein.

### Cryo-Electron Microscopy (Cryo-EM) Measurements

All samples used in cryo-EM measurements were incubated, with or without αS (200 μM), for 1 h at 298K using fresh preparations of SUV-31% at a concentration of 0.05%. The SUV concentrations were calculated by considering exclusively the DOPE:DOPS:DOPC component. After incubation cryo-EM grids were prepared by vitrifying the sample solutions using aliquots of 2 μL and a Vitrobot Mark III apparatus (SMIF, Duke University, Durham, United Kingdom) at a relative humidity of 100% at 20°C. The samples were loaded on a glow-discharged copper Quantifoil R2/2 grid (Quantifoil GmbH, Germany) and blotted with filter paper for 2.5 s to leave a thin film of solution. The blotted samples were immediately plunged into liquid ethane and stored under liquid nitrogen prior to imaging. Micrographs were aquired using a Tecnai T12 twin (LaB_6_) electron microscope operating at 120 kV (FEI, Hillsboro, Oregon, United States), using a Gatan 626 cryo-holder (Gatan, Pleasanton, United States) cooled with liquid nitrogen to temperatures below -170°C. Digital micrographs were acquired on a TVIPS F216 CCD camera using the EMMENU 4 software package (TVIPS, Munich, Germany).

## Results

### Influence of Cholesterol on the Binding Affinity of αS With Synaptic-Like SUVs

We used synaptic-like small unilamellar vesicles (SUVs) composed of a mixture of 1,2-dioleoyl-sn-glycero-3-phosphoethanolamine (DOPE), 1,2-dioleoyl-sn-glycero-3-phospho-L-serine (DOPS), 1,2-dioleoyl-sn-glycero-3-phosphocholine (DOPC) at a ratio w/w of 5:3:2 (Bodner et al., 2009). This mixture has been widely used to study the binding of αS with synaptic membranes (Bodner et al., 2009, 2010; Maltsev et al., 2012; Ouberai et al., 2013; Fonseca-Ornelas et al., 2014; Fusco et al., 2014, 2016b; Ysselstein et al., 2015), and is considered to be a good model of SV membrane in terms of curvature, charge and lipid composition (Bodner et al., 2009). A second type of SUVs was prepared using DOPE:DOPS:DOPC lipids (5:3:2 w/w) and cholesterol, the latter amounting to 31% w/w of the total mixture to mimic the natural abundance in the membrane component of SVs (Takamori et al., 2006). The two types of vesicles are denoted as SUV-0% and SUV-31% throughout this article.

We first characterized the binding affinity using CD to probe the overall conformational transition of αS upon interaction with the SUVs. In aqueous solution, the CD spectrum of αS is typical of a random coil protein, whereas upon incubation with acidic SUVs the spectrum adopts the characteristic shape measured for α-helical proteins (Fusco et al., 2014). We performed CD measurements of αS in the presence of different concentrations the SUVs to probe the transition from disorder-unbound to α-helical-bound states of the protein, as monitored at 222 nm, [θ]_222_. The data indicate that cholesterol affects the binding properties of αS, resulting in weaker interactions with SUV-31% than SUV-0% (Figures 1B,C). We used the Hill equation to fit the CD titrations and to calculate an apparent dissociation constant, *K*_*D*_:

$$\chi_{B}=\frac{{\left[L\,i\,p\,i\,d\right]}^{n}}{K_{D}+{\left[L\,i\,p\,i\,d\right]}^{n}}$$

where χ_*B*_ is the fraction of bound helix probed at [θ]_222_, *K*_*D*_ is the apparent dissociation constant, and *n* is the Hill coefficient describing the cooperativity of the process. The results indicate that both vesicle compositions here analyzed are bound by αS with a significant level of cooperativity, as indicated by *n* coefficients larger than 1 (2.11 ± 0.14 and 2.03 ± 0.17 for SUV-0% and SUV-31%, respectively). By contrast, the apparent binding constants *K*_*D*_ indicate that the membrane affinity of αS is 2.6 times stronger for SUV-0% (0.376 ± 0.03 mM) than SUV-31% (0.972 ± 0.09 mM, Figures 1D,E). The effect of cholesterol was further investigated by performing CD titrations made with intermediate concentrations of the molecule in the SUV mixture, namely 10% and 20% w/w. Overall, a linear correlation was found between the amount of cholesterol and the resulting *K*_*D*_ within the range of 0–31% w/w (Supplementary Figure S1).

It is worth noting that in SUV-31% the fraction of charged components (DOPS) in the total mixture is reduced because of the presence of an additional non-charged component (cholesterol). Since negative charges play a key role in membrane binding by αS, we decoupled the charge effect from the results of our affinity measurements. This was achieved by considering only the DOPE:DOPS:DOPC component in the calculation of the SUV concentrations, which provided consistent αS:DOPS ratios in the CD titrations made with the two types of vesicles. Indeed, the inclusion of cholesterol in the calculation of SUV-31% concentrations would result in significantly weaker binding affinities as a consequence of the lower αS:DOPS ratios (Supplementary Figure S2). In order to further investigate the charge effect associated with the presence of cholesterol in the lipid mixture, we measured the affinity of αS for modified SUV-31% in which cholesterol is substituted by another non-charged component (POPE) (Supplementary Figure S3). The results show a binding affinity that is significantly stronger than that of SUV-31% and very close to that of SUV-0%. This result indicates that the charge effect is not a determining factor in the low affinity of SUV-31%.

### Structural Properties of αS Bound to Synaptic-Like SUVs Containing Cholesterol

We then used CEST in solution NMR spectroscopy (Milojevic et al., 2007; Huang et al., 2008; Fawzi et al., 2011; Vallurupalli et al., 2012; Fusco et al., 2014; Romano et al., 2018) to probe the equilibrium between membrane bound and unbound states of αS at the resolution of individual amino acid residues. CEST has been used to accurately characterize the equilibrium between membrane unbound (detectable in solution-NMR spectroscopy) and membrane bound (undetectable) states of αS (Fusco et al., 2014, 2016b). By applying a continuous weak radiofrequency field off-resonance (up to ± 28 kHz) in the ^15^N channel, it is possible to selectively saturate the membrane-bound state of the protein (Milojevic et al., 2007; Huang et al., 2008; Fawzi et al., 2011; Vallurupalli et al., 2012; Fusco et al., 2014; Romano et al., 2018). This saturation is then transferred *via* chemical exchange to the unbound state and detected as an attenuation of the signal intensities of the protein residues, thereby directly probing the strength of membrane interaction.

**FIGURE 2:**
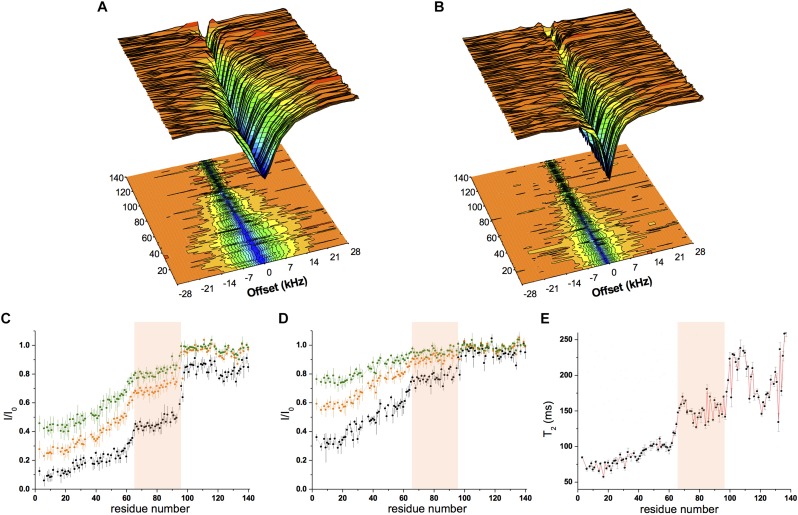
Conformational properties of α*S* binding to acidic SUVs. **(A–D)** CEST experiments were recorded at a ^1^H frequency of 700 MHz (see section “Materials and Methods”), using a protein concentration of 300 μM and 0.06% (0.6 mg/ml) of SUV-31%. The concentration of SUV-31% was calculated by considering exclusively the DOPE:DOPS:DOPC component of the lipid mixture. ^1^H-^15^N CEST spectra were recorded by using a continuous wave saturation (170 Hz or 350 Hz) on the ^15^N channel at a range of offsets: −28, −21, −14, −9, −5, −3, −1.5, 0, 1.5, 3, 5, 9, 14, 21, and 28 kHz. An additional spectrum saturated at –100 kHz was recorded as a reference. CEST surface was measured using a saturation bandwidth of 350 Hz **(A)** and 170 Hz **(B)** and the saturation along the αS sequence (350 Hz and 170 Hz in panels **(C,D)**, respectively) is shown. Black, orange and green lines refer to the averaged CEST profiles measured using offsets at ± 1.5 kHz, ± 3.0 kHz, and ± 5.0 kHz, respectively. **(E)** *T*_2_ values from transverse relaxation measurements [experimental conditions as in panels **(A–D)**]. The pink background in panels **(C–E)** evidences region 65–97, where a reduction in binding compared to the preceding segment is observed in both CEST (reduced saturation) and relaxation (higher *T*_2_ values) measurements.

**FIGURE 3:**
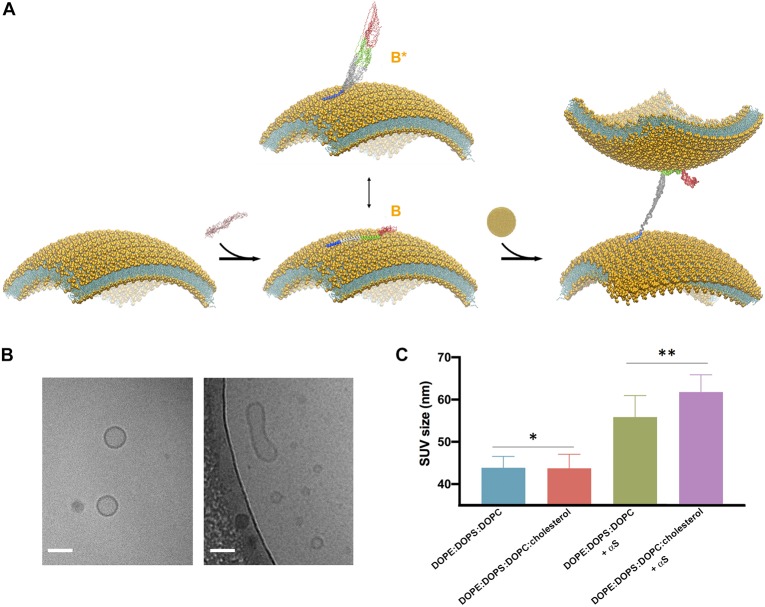
Cholesterol enhances vesicle-vesicle interactions promoted by αS. **(A)** Schematic representation of the SUV binding modes of αS and the double-anchor mechanism for vesicle-vesicle interactions (Fusco et al., 2016b). Upon binding to acidic SUVs, αS adopts an ensemble conformation (center). Two major conformational states are shown in the scheme, namely a fully α-helical state spanning the whole region 1–97 (state B) bound to the same membrane and a state B^∗^ that interacts with the membrane exclusively through the N-terminal anchor (residues 1–25), with the rest of the protein being detached from the membrane surface. The state B^∗^ is an active conformation for the recruitment of a second vesicle through the double-anchor mechanism (Fusco et al., 2016b) (right), with a single αS molecule binding two vesicles *via* its N-terminal region (lower vesicle) and the region 65–97 (upper vesicle). The population of B^∗^ measured using CEST experiments was found to increase from 38% to 51% when interacting with SUV-0% and SUV-31%, respectively. Color codes for the regions of αS are blue (1–25), gray (26–64), green (65–97), and red (98–140). **(B)** Examples of isolated (left) and fused (right) SUV-31%, as probed from images obtained *in vitro* by cryo-EM, the scale bar is 50 nm. **(C)** DLS measurements of SUVs, either isolated or incubated with αS. Samples used in DLS measurements were incubated, with or without αS molecules, for 1 h at 298 K using freshly prepared SUVs at a concentration of 0.05% and made with SUV-0% and SUV-31%. The SUV concentrations were calculated by considering the DOPE:DOPS:DOPC component exclusively in both types of vesicles. In order to compensate for the 2.6 difference in *K*_*D*_, the concentrations of αS when incubated with SUV-0% and SUV-31%, were 77 μM and 200 μM, respectively, which enables to decouple the differences in the binding affinities from the tendency to promote vesicle fusion. Each measurement was made of 10 replicates. Error bars report standard deviations of the centers of the size distributions in the 10 replicates. The single (*) and double (**) asterisks indicate *P* = 0.9264 and *P* = 0.01, respectively. These were calculated using the unpaired *t*-test with Welch’s correction (Sawilowsky, 2002).

Chemical exchange saturation transfer was measured using a sample composed of αS (300 μM) incubated with SUV-31% (0.6 mg/ml) with two different continuous-wave radio frequencies (170 Hz and 350 Hz, Figures 2A,B). The data showed strong saturation in the N-terminal region of the protein, indicating significant affinity for lipid membranes, whereas saturation then gradually decreased from residue 26–97, indicating a progressive reduction in the membrane affinity of the central region of αS (Figures 2A–D). Finally, the C-terminal segment (98–140) resulted very weakly associated with the membrane as shown by very low CEST saturation values. By comparing these results with CEST profiles previously measured using SUV-0% (Fusco et al., 2014), the only significant difference in the two measurements was found in the region spanning residues 65–97, with weaker local binding for acidic SUVs containing cholesterol (Figures 2C,D). We analyzed the CEST saturations measured using a bandwidth of 350 Hz at offsets of ± 1.5 kHz to provide a quantitative estimation of the population of states in the membrane-bound αS featuring the detachment of the region 65–97 from the lipid bilayer (state B^∗^ in Figure 3A) in contrast to conformations featuring a fully bound region 65–97 (state B in Figure 3A). The data indicate that the population of B^∗^ states is 38%, when the αS interacts with SUV-0% and rises to 51% in the case of SUV-31%. The shift toward unbound populations in the region 65–97 is consistent with transverse relaxation measurements (Figure 2E) showing high *T*_2_ values for this region upon interaction with SUV-31%.

### Influence of Cholesterol on the Vesicle-Vesicle Interactions Promoted by αS

Our previous study based on the familial mutations of αS indicated that the membrane binding modes of the region 65–97 and of the N-terminal anchor have some degree of independence, leading to the suggestion that, in addition to interacting with a single membrane surface, these two regions can also bind simultaneously two different vesicles and induce their interaction (Fusco et al., 2016b; Figure 3A). This “double-anchor” mechanism was confirmed by a mutant of αS designed to have higher efficiency in mediating vesicle-vesicle interactions as a result of an enhanced exposure of the region 65–97, as observed both using synaptic-like SUVs and *ex vivo* SVs (Fusco et al., 2016b). This evidence indicated that the detachment of the region 65 to 97 from the membrane surface is a dominant factor to promote αS-induced vesicle-vesicle interactions, and overcomes the loss of local membrane binding affinity. In accordance with the double-anchor mechanism, the deletion of the segment 71–82 and the weakening of the membrane affinity of the N-terminal anchor region were found to impair vesicle clustering by αS in *S. cerevisiae* (Soper et al., 2008).

Since the present NMR results indicate a weaker association of the region 65–97 to cholesterol-containing SUVs, we tested the promotion of clustering of these vesicle induced by αS. When synaptic-like SUVs are used *in vitro*, vesicle-vesicle interactions promoted by αS result in vesicle fusion (Bodner et al., 2009; Fusco et al., 2016b; Figure 3B), a process that can be monitored both at a single vesicle level or in bulk measurements (Fusco et al., 2016b). We used here a previously optimized protocol (Fusco et al., 2016b) based on bulk experiments of DLS to monitor how the average size of SUV-0% and SUV-31% is affected by the interaction with αS (Figure 3C). In estimating vesicle fusion promoted by αS, DLS has proved to be an accurate and fast method, generating results that are in agreement with super-resolution STED imaging but with the advantage of performing measurements in bulk solution (Fusco et al., 2016b). In the absence of αS the two types of vesicles were prepared with the same average size (43.9 ± 2.7 nm and 43.7 ± 3.3 nm for SUV-0% and SUV-31%, respectively, *P* value of 0.93). The sizes of both vesicle samples, however, increased when incubated with αS (56 ± 5 nm and 62 ± 4 nm for SUV-0% and SUV-31%, respectively, *P* value of 0.01). These data therefore confirm the previous observations obtained with SUV-0% (Fusco et al., 2016b) and also indicate that the presence of cholesterol alters the conformation of the membrane-bound αS as well as its ability to induce the interaction and fusion of acidic vesicles containing cholesterol. This effect is likely to arise from an enhanced population of B^∗^ states at the surface SUV-31%, which is the active conformation for the double-anchor mechanism promoting vesicle-vesicle interactions (Figure 3A). A similar effect was indeed previously observed using a designed mutant of αS that promotes enhanced detachment of the region 65–97 from the surface of SUV-0% as well as stronger vesicle-vesicle interactions (Fusco et al., 2016b).

## Discussion

In this work, we have investigated how cholesterol affects the equilibrium between cytosolic and membrane-bound states of αS. This equilibrium is crucial for the physiological properties of this protein (Fusco et al., 2018), as well as the kinetics of αS aggregation (Perrin et al., 2001; Necula et al., 2003; Sharon et al., 2003; Zhu and Fink, 2003; Auluck et al., 2010; Comellas et al., 2012) and the toxicity of the resulting aggregates (Winner et al., 2011; Lorenzen et al., 2014; Fusco et al., 2017). Cholesterol is a key component of the membrane of SVs (Takamori et al., 2006), and is expected to influence the membrane binding of αS. We therefore aimed at characterizing the membrane interaction of αS under conditions resembling as close as possible the physiological properties of SV membranes. The relationship between cholesterol and PD is highly debated in the literature (Bosco et al., 2006; Liu et al., 2010), with several observations indicating a link between αS and the levels of cholesterol in cellular membranes (Fantini et al., 2011; van Maarschalkerweerd et al., 2014; Hsiao et al., 2017). Cholesterol was shown in particular to enhance the propensity of αS to induce the formation of defects in lipid bilayers (Pan et al., 2018), whereas αS can remodel cholesterol-enriched ternary membranes that mimic cellular raft-like domains (Leftin et al., 2013).

Our data indicate that the presence of cholesterol in the lipid bilayer reduces the overall binding affinity for synaptic-like vesicles, a result in agreement with studies in yeast showing that the inhibition of sterol synthesis enhances the vesicular localization of αS (Valastyan et al., 2014). A detailed analysis based on NMR CEST and relaxation experiments indicated that the structural effect of cholesterol on the membrane interaction by αS is restricted to residues in the NAC region, indicating a link between the overall binding affinity of the protein and the local interactions of the region 65–97. Similarly, the high binding affinity of αS for POPG SUVs, three times stronger than the binding with SUV-0%, was attributed to the strong local interaction of the central region of the protein (Fusco et al., 2014). Taken together these data support the emerging idea that the central region of αS plays the role of a membrane sensor that determines the overall binding affinity for lipid membranes. We also found that the double-anchor mechanism, which promotes vesicle-vesicle interactions by αS, is enhanced in the presence of cholesterol, thus providing new evidence that the exposure of the region 65–97 in the membrane-bound state of αS is crucial for inducing vesicle clustering (Fusco et al., 2016b). In addition to the possibility that a broken α-helix structure, which is a conformation that αS adopts upon binding with detergent micelles (Ulmer and Bax, 2005; Mazumder et al., 2013), can be involved in the promotion of vesicle-vesicle interactions (Dikiy and Eliezer, 2012; Snead and Eliezer, 2014), our data indicate that the balance between membrane-bound (ordered) and membrane-detached (disordered) species in the region 65–97 is indeed the crucial modulator of the ability of αS to bridge multiple vesicles and induce their clustering. It is worth noting that our work focuses specifically on the αS-membrane interaction, however, protein-protein interactions at the surface of SV, including binding with VAMP-2 (Burre et al., 2010; Diao et al., 2013) and Rab3a (Chen et al., 2013), are also crucial for the regulation of SV trafficking. The binding of αS with the membrane and protein components of SV can have cooperative effects, as shown in studies in synaptosomes, where high concentrations of Ca^2+^ were correlated with both an enhanced double-anchor mechanism and a strong colocalization with VAMP-2 (Lautenschlager et al., 2018).

The highlighted region 65–97 of αS is also known to span most of the NAC sequence, a region that has been largely associated with the mechanisms of αS aggregation (Cookson, 2005; Chiti and Dobson, 2006; Rodriguez et al., 2015). The present study provides further evidence that the NAC region may not only act as the most amyloidogenic region of αS, but also have a functional relevance. Perturbations in the local membrane interactions of residues in the NAC, as we have observed here as a result of the presence of cholesterol in the lipid bilayer, can significantly alter the properties of the membrane-bound state of αS, including its overall binding affinity and the ability to induce vesicle-vesicle interactions.

In conclusion our study has identified the structural basis of the modulation of the membrane binding affinity of αS by cholesterol. We found that the fine-tuning between bound and unbound conformations in the NAC region is a key element modulating the behavior of αS at the surface of synaptic-like membranes. Perturbations of this balance are expected to generate aberrant protein-protein interactions leading to membrane-induced aggregation (Auluck et al., 2010; Grey et al., 2011, 2015; Comellas et al., 2012) or to impair some functional conformations that play a role in the normal physiological state of αS.

## Data Availability Statement

All datasets generated for this study are included in the article/Supplementary Material.

## Author Contributions

GF, AD, and CD conceived the experiments. WM, AD, JB, and GF conducted the experiments. All authors analyzed and discussed the results. AD, MV, and GF wrote the manuscript with input from all authors.

## Conflict of Interest

The authors declare that the research was conducted in the absence of any commercial or financial relationships that could be construed as a potential conflict of interest.
