# Supplementary material for: A Role of Cholesterol in Modulating the Binding of α-Synuclein to Synaptic-Like Vesicles
Source: Front Neurosci. 2020 Jan 29;14:18. doi: 10.3389/fnins.2020.00018 (PMC7000551; doi:10.3389/fnins.2020.00018)
Supplement: Supplementary file 1 [file Data_Sheet_1.PDF]

Supplementary figure 1.

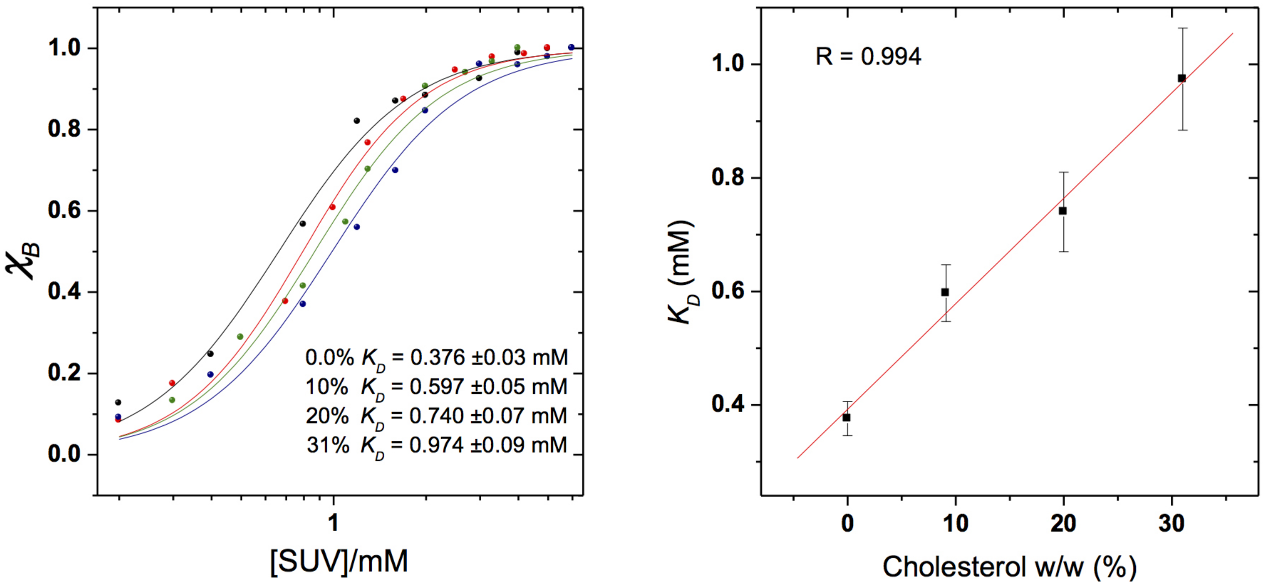

Figure S1. Effect of cholesterol in the binding affinity of αS with acidic SUVs. CD titrations were performed with different concentrations of cholesterol. The data shows binding curves for SUV-0%, SUV-10%, SUV-20% and SUV-31%. The concentrations of the SUVs were calculated by considering exclusively the DOPE:DOPS:DOPC component in all types of vesicles. Fitting of the CD titrations were based on the signal at 222 nm, $[\theta]_{222\ nm}$, using the Hill equation to account for both binding constant K_D and the cooperativity (Hill coefficient n). The analysis shows a strong linear correlation between K_D values and amount of cholesterol (w/w) in the lipid mixture.

Supplementary figure 2.

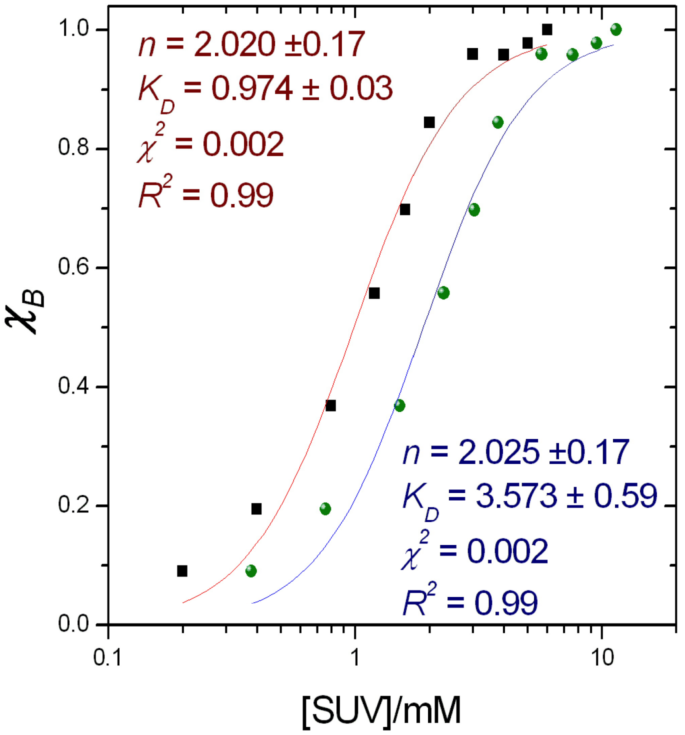

Figure S2. Binding curve of αS with SUV-31%. Two different analyses of the same CD titration are shown. In the first case, the SUV concentration was calculated using exclusively the DOPE:DOPS:DOPC component (red fitting), in order to provide the same αS:DOPS ratios as used in SUV-0%. In the second case the SUV concentration was calculated by considering the whole mixture DOPE:DOPS:DOPC:cholesterol (blue fitting). In this case, the dilution of the charged component (DOPS) of the mixture would lead to a much weaker binding affinity and a binding curve that cannot be compared with that measured with SUV-0%.

Supplementary figure 3.

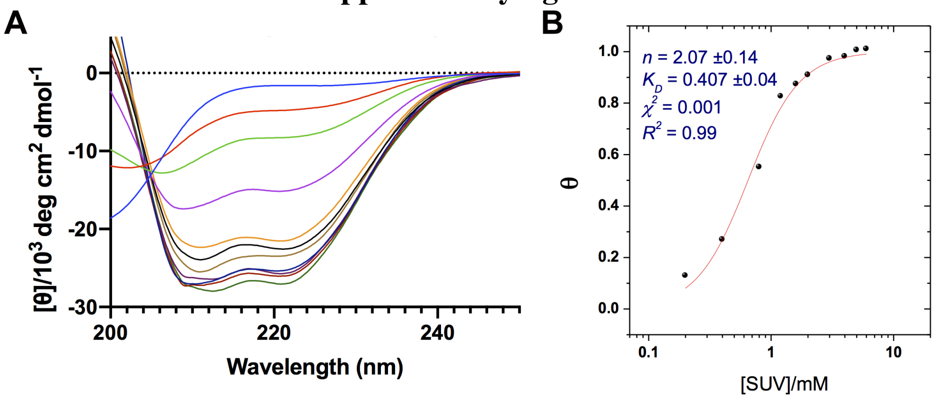

Figure S3. Binding curve of αS with SUV composed of DOPE:DOPS:DOPC:POPE. In order to further assess the charge effects on the affinity of αS to bind SUV-0% and SUV-31%, we performed a CD titration using a modified SUV-31% mixture in which cholesterol was substituted with another non-charged component (POPE) at the same molar ratios (DOPE:DOPS:DOPC:POPE in molar ratios 5:3:2:9 instead of DOPE:DOPS:DOPC:Cholesterol in molar ratios 5:3:2:9). The results provide evidence for a binding affinity for this mixture (0.407 ± 0.04 mM) that is very close to the one obtained using SUV-0% (0.376 ± 0.03 mM) and significantly stronger than the one obtained with SUV-31% containing cholesterol (0.972 ± 0.09 mM). These data, therefore, attribute the lower affinity of αS for SUV-31% to presence of cholesterol and not to the charge effect associated with a fourth neutral molecule in the lipid mixture.
